# Effect of MT2A on apoptosis and proliferation in HL60 cells

**DOI:** 10.7150/ijms.57821

**Published:** 2021-06-04

**Authors:** Yu-Qing Pan, Min Niu, Shu-min Liu, Yu-Xia Bao, Kai Yang, Xiao-Bo Ma, Liang He, Yi-Xun Li, Jie-Xian Cao, Xi Zhang, Yan Du

**Affiliations:** 1 Department of Clinical Laboratory, the First Affiliated Hospital of Kunming Medical University, Kunming, Yunnan, P.R. China.; 2 Yunnan Key Laboratory of Laboratory Medicine, Kunming, Yunnan, P.R. China.; 3 Yunnan Innovation Team of Clinical Laboratory and Diagnosis, the First Affiliated Hospital of Kunming Medical University, Kunming, Yunnan, P.R. China.; 4 Department of Clinical Laboratory, Yunnan Cancer Hospital, the Third Affiliated Hospital of Kunming Medical University, Kunming, Yunnan, P.R. China.

**Keywords:** acute myeloid leukemia, MT2A, apoptosis, proliferation.

## Abstract

Although accumulating evidence has revealed that metallothioneins (MTs) and its family member MT2A are strongly linked to the risk of various solid tumors, researches on the occurrence and development of acute myeloid leukemia (AML) have rarely been investigated. Here, we constructed a lentiviral vector with MT2A over-expression and the interfering plasmids with MT2A expression inhibition to study the influence of MT2A on the bioactivities of HL60 cells. After cells were infected with a lentiviral vector containing the MT2A gene, both transcription and translation levels of MT2A were significantly increased in the over-expressed group in comparison with control groups. In vitro experiments, all results demonstrated that cell reproductive capacity was inhibited, but cell apoptosis rate was significantly increased. Together, the expression of apoptosis-related protein Bcl2 was remarkably reduced, while a high expression level of Bax protein was detected. Further experiments revealed that up-regulation of MT2A induced cell apoptosis and promoted G2/M phase arrest. The mechanism may be associated with down-regulated p-IκB-α and cyclinD1 expression and up-regulated IκB-α expression in the nuclear factor-kappaB (NF-κB) pathway. On the contrary, MT2A expression was down-regulated by interfering plasmids. We found that cell proliferative potential was notably increased in the interfering group compared with the negative and untreated group. What's more, MT2A may be closely related to AML cell proliferation and function via the NF-κB signal pathway.

## 1. Introduction

Leukemia, a group of hematologic malignancies, is derived from hematopoietic stem and progenitor cells. Clinically, due to uncontrolled proliferation of leukemia cells, normal hematopoietic suppression of bone marrow and infiltration of extramedullary tissues and organs, it will cause anemia, infection, hemorrhage, hepatosplenomegaly, and even diffuse endovascular clotting and other symptoms 1. Statistics show that the total incidence of AML has worldwide increased from 18% in 1990 to 23.1% in 2017 2. However, the prognosis of AML patients has improved significantly due to improved treatment and other protocols. Among them, the prognosis of children with AML is generally better than that of adults and adolescents, and the long-term survival rate is close to 70% 3, 4. Recurrent genetic abnormalities of this disease typically include fusion gene AML1-ETO in t (8;21) (q22; q22) leukemia, PML-RARα in t (15;17) (q22; q12), CBFB-MYH11 in inv (16) (p13.1 q22)/t (16;16) (p13.1; q22), and AML with 3q, 11q23 , -5/del(5q), -7/del(7q)^5^. Plenty of clinical trials based on the understanding of genetic abnormalities and prognosis of AML have shown that patients with t (8;21), t (15;17) and inv (16)/t (16;16)/del (16) have a good prognosis 6.

However, the AML patients with an unfavorable prognosis, extraordinarily complex karyotypes consisted of -5/del(5q), -7/del(7q), and 11q23, have become an international consensus 7. Traditionally, AML patients are mainly treated with cytarabine/anthracycline ("7+3") combined with Gituzumab intensive chemotherapy protocols 8, and azacarbine/decitabine/venetoclax combined with hypomethylated chemotherapy drugs or low-intensity chemotherapy with low dose cytarabine^9^. In fact, due to drug reactions and resistance to cancer chemotherapy, new targeted drugs including Ivosidenib and Enasidenib failed to achieve complete remission in ~40% refractory/relapsed AML patients. Besides, a legacy of stubbornly high costing has further limited progress in leukemia treatment 10. Also, owing to the lacking of a suitable donor for hematopoietic stem cell transplantation, the therapeutic effect is still not satisfactory. Of particular note is that there is no significant improvement in early hemorrhagic mortality of acute promyelocytic leukemia (APL). Therefore, retinoids are not effective against all APL, and sometimes their toxic and side effects are even fatal 11. Hence, there is an urgent need to investigate the pathological mechanisms of AML, even to provide potential critical therapeutic targets for the study of AML. With a highly conserved low molecular weight protein character, MTs make a critical difference in detoxification and binding metal ion 12. In recent years, studies have shown that MTs is differentially expressed in colorectal cancer 13, hepatocellular carcinoma 14 and prostate cancer 15, suggesting that MTs may be closely related to the progression of some solid tumors. Besides, MTs play an important role in the evolution and survival of tumor cells by regulating the smooth transition of the cell cycle 16. Therefore, we speculated that MTs maybe serve as regulatory factors act a pivotal physiological role in the proliferation of tumor cells. The protein MT2A consisted of 61 amino acids, is peculiarly prone to combine with heavy metals 17. Researches show that MT2A have a different prognosis in different types of tumors depending on the tissue or cell type 18. Therefore, we believed that abnormal expression of MT2A in tumor cells could affect cell survival outcome.

In the current study, to identify MT2A's “gain or loss of function” on the biological function of HL60 cell, we performed a series of molecular biological methods to clarify the function of MT2A in the etiology of AML. Besides, our research results provided a practical theoretical basis and data support for an in-depth understanding of the molecular mechanism and pathogenic signaling pathway of AML as well as its precision treatment and prognosis assessment.

## 2. Materials and methods

### 2.1. AML cells line

HL60 cells (ATCC, Rockville, MD, USA) were inoculated in RMPI-1640 medium (Gibco, MD, USA) supplemented with streptomycin (0.1g/L, Beyotime Biotechnology, Shanghai, China), penicillin (100 U/L, Beyotime Biotechnology, Shanghai, China) and 12% fetal bovine serum (Gibco, MD, USA) at 37℃ and containing 5% CO2. And the cells in good growth state, pollution-free and in logarithmic growth stage were selected for subsequent experiments.

### 2.2. Construction of recombined interfering plasmid and lentiviruses

A total of three short hairpin RNA (shRNA) targeted to MT2A mRNA sequence (NM_005953.5), and one negative control vector were designed and synthesized by Shanghai GenePharma Co., LTD. Then an optimal infection sequence was selected in vitro for subsequent experiments. The sequences of each interference and control group are as follows:

psiMT2A-1: Sense:5'-GATCCGCTCCCAGATGTAAAGAACGCTTCAAGAGAGCGTTCTTTACATCTGGGAGCTTTTTT-3'; Antisense:5'-GAAGACAAAAAAGCTCCCAGATGTAAAGAACGCTCTCTTGAAGCGTTCTTTACATCTGGGAGC-3';

psiMT2A-2: Sense:5'-GATCCGATGTAAAGAACGCGACTTCCTTCAAGAGAGGAAGTCGCGTTCTTTACATCTTTTTT-3'; Antisense:5'-GAAGACAAAAAAGATGTAAAGAACGCGACTTCCTCTCTTGAAGGAAGTCGCGTTCTTTACATC-3';

psiMT2A-3: Sense:5'-GATCCTTCCTTTTTCTATGAAATAATGTTTCAAGAGAACATTATTTCATAGAAAAAGGAATTTTTT-3'; Antisense:5'-GAAGACAAAAAATTCCTTTTTCTATGAAATAATGTTCTCTTGAAACATTATTTCATAGAAAAAGGAA-3';

Negative control: Sense:5'-CACCGTTCTCCGAACGTGTCACGTCAAGAGATTACGTGACACGTTCGGAGAATTTTTTG-3'; Antisense:5'-GATCCAAAAAATTCTCCGAACGTGTCACGTAATCTCTTGACGTGACACGTTCGGAGAA-3'. In addition, lentivirus GV492-MT2A and GFP-expressed lentivirus vector without MT2A gene (GV492-KZ) were constructed by Shanghai Genechem Co., LTD.

### 2.3. Cell transfection

The 6-well plates were used to inoculate HL60 cells with a concentration of ~1×10^6^ cells/well.

The interfering plasmid psiMT2A-1, psiMT2A-2, and psiMT2A-3 and the negative control plasmid were transfected into HL60 cells based on the directions of Lipofectamine™ 3000 (Invitrogen, Carlsbad, California, USA). The green fluorescent protein (GFP) expression in HL60 cells was observed by fluorescence microscope, and the transfection efficiency was determined by FCM. And methods to set up a negative control group (NC siRNA), untreated group (HL60 cells only), and interfering group (psiMT2A-1, psiMT2A-2, and psiMT2A-3).

### 2.4. Cell infection by lentivirus

The cell suspension with a density of 10×10^5^/mL was manufactured using DMEM medium (HyClone, MD, USA) and inoculated into a 6-well plate with 40 μL of lentivirus (1×10^6^ TU/ml) and 16 μL of 25× HiTtansG (Genechem, Shanghai, China). At 48 h of infection, the cells were replaced with medium to maintain cell activity. The infection efficiency was observed by fluorescence microscopy (Nikon, Tokyo, Japan) at 96 h after infection.

### 2.5. CCK‑8 assay

For research purposes, 96-well plates were performed to inoculate cells at a density of 5×10^3^ cells/well, and each group was provided with five multiple wells. Then the plates were incubated for 12~72h at 37 ℃ with 5% CO_2_. We next added 10 µl CCK-8 solution (5 mg/ml, Abbkine, Biotechnology, Wuhan, China) to each well and detected the absorbance at 450 nm by spectrophotometer (Bio-Rad Laboratories, CA, USA). Finally, the smooth cell growth curve was made, and the experiment was repeated three times.

### 2.6. Cloning and formation experiment of soft agar

Cells in each group (200cells/well) were added to a 24-well plate containing 0.9% methylcellulose, and four parallel controls were set in each group. After continuous culture for ten days, colony counting (greater than or equal to 50 cells) was achieved by the inverted microscope (Nikon, Tokyo, Japan).

### 2.7. qPCR assay

The total RNA extraction for each group was implemented by TRIzol (Gibcobrl, CA, USA), and determination of RNA concentration was executed by spectrophotometer (Multiskan Sky, ThermoFisher, CA, USA). And then, total RNA with the absorbance of 1.8 ~2.0 was selected for synthesis of cDNA (ApexBio Technology, Thermo Fisher Scientific, Inc., TX, USA). Moreover, the gene-specific sense and antisense primer of qPCR, which targeted the MT2A and GAPDH, were designed by Sangon Biotech, Inc., China. Primers for MT2A (Invitrogen, Carlsbad, California, USA) were; forward, 5'-ATGGATCCCAACTGCTCCTG-3' and reverse, 5'-AGCAGCAGCTTTTCTTGCAG-3'. GAPDH, forward, 5'-TTGTCATGGGAGTGAACGAGA-3' and reverse, 5'-CAGGCAGTTGGTGGTACAGG-3'. Besides, cDNA was subjected to templates for qPCR assays, and 25 µl of reaction mix included 10 µl of 2×TB Green Fast Mix (Takara Bio Inc., CA, USA), 1 µl of each primer (10 µM), 1 µl cDNA, 13 µl RNase free water. An initial amplification was performed with pre-degeneration (90 ℃, 3 min), denaturation (90 ℃, 30 sec), anneal process (60 ℃, 35 sec), and extension process (72 ℃, 30 sec), 38 cycles. The number of experiment repetitions was strictly controlled at least three times, together with the 2^‑ΔΔCt^ method to measure the relative quantitative of gene expression 19.

### 2.8. Western blot analysis

The extracted cellular proteins were separated with 10% SDS-PAGE, and the equivalent amount of sample was kept for each well. Then, polyvinylidene fluoride membranes (EMD Millipore, Billerica, MA, USA) was used to transfer the target protein. And membrane blocking process was performed by 5% skimmed milk for 2 hours at room temperature and tested by immunoblotting with the BeyoECL Plus (Beyotime Biotechnology, Shanghai, China). Primary antibodies were as follows: anti-human MT2A/IκB-α mouse monoclonal antibody(diluted 1:2000, Cat.No. ab12228/ab12134, Abcam, Cambridge, UK), anti-human Bax/Bcl2 mouse monoclonal antibody (diluted 1:500, Cat.No. AF0054/AF6285, Beyotime Biotechnology, Shanghai, China), anti-human CyclinB1/CDK1/CyclinD1/p-IκB-α(ps36) rabbit monoclonal antibody(diluted 1:2000, Cat.No. ab215436/ab133327/ab16663/ab133462, Abcam, Cambridge, UK), anti-human p65 rabbit monoclonal antibody (diluted 1:1000, Cat.No. 8242, Cell Signaling Technology, MA, USA).

### 2.9. FCM assay

The treated cells in each group were washed twice with phosphate-buffered saline (PBS, Beyotime Biotechnology, Shanghai, China), then fixed with 75% ethanol at 4 °C overnight, and stained with fluorescent dye for 40 min. Flow cytometry (Beckman Coulter, CA, USA) was adopted to measure apoptotic cell rate and cell cycle change.

### 2.10. Statistical analysis

All statistical analysis was performed with SPSS 23.0 tool (SPSS, Inc., IL, USA). The one-way ANOVA with the least significant difference (LSD) test was applied to compare the difference of multiple parametric data sets, and data of three independent tests were represented as the mean ± SD. *P* < 0.05 for the difference was statistically significant.

## 3. Results

### 3.1. Analysis of MT2A knock-down effect by qPCR and Western blot

**Figure 1:**
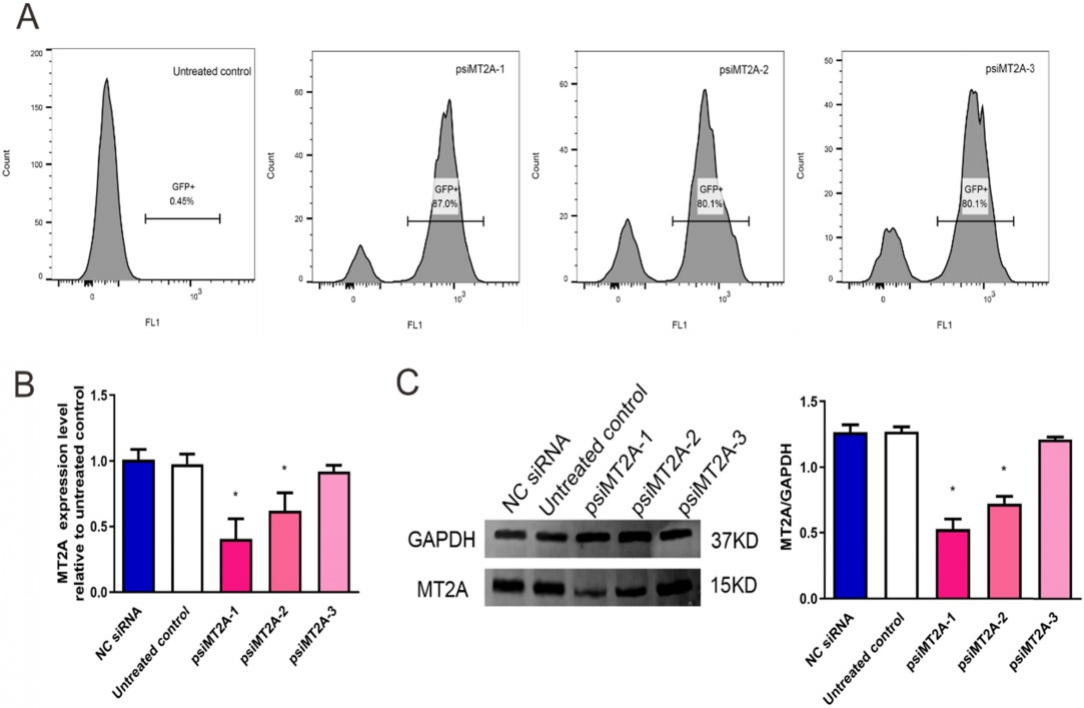
** HL60 cell transfection induces down-regulation of MT2A.** (A) FCM analysis of transfection efficiency in the untreated, psiMT2A-1, psiMT2A-2, and psiMT2A-3 group at 72 h post-transfection. (B) qPCR analysis of MT2A expression in each group at mRNA level. GAPDH was used to normalized gene expression. mRNA expression in the psiMT2A-1 group was remarkably down-regulated compared with the control group. (C) Western blot analysis of MT2A down-expression in each group at the protein level. Relative MT2A protein level was quantitatively evaluated by densitometric analysis. The number of independent experiment repetitions was tightly controlled at least three times(right) and represented as mean ± SD. *P < 0.05 vs. control.

The results demonstrated that transfection efficiency of psiMT2A-1, psiMT2A-2, and psiMT2A-3 was 87%, 80.1%, and 80.1%, respectively, while the untreated group was 0.45% due to the absence of interference plasmid (Figure 1A). Moreover, the mRNA expression level of MT2A was notably decreased after transfection of the short hairpin RNA (shRNA) psiMT2A-1 and psiMT2A-2 in comparison with the untreated group (*P* < 0.05, Figure 1B). However, the psiMT2A-3 group showed no statistically significant difference (*P*=0.29, Figure 1B). Besides, the expression level of MT2A protein in both the psiMT2A-1 and the psiMT2A-2 group was notably reduced in comparison with the untreated group. Still, the decreasing tendency became more pronounced in the psiMT2A-1 group (*P* < 0.05, Figure 1C). No statistical difference was found in the expression level of MT2A protein for the psiMT2A-3 group (*P*=0.19, Figure 1C).

### 3.2. Down-regulation of MT2A expression in HL60 cell growth

**Figure 2:**
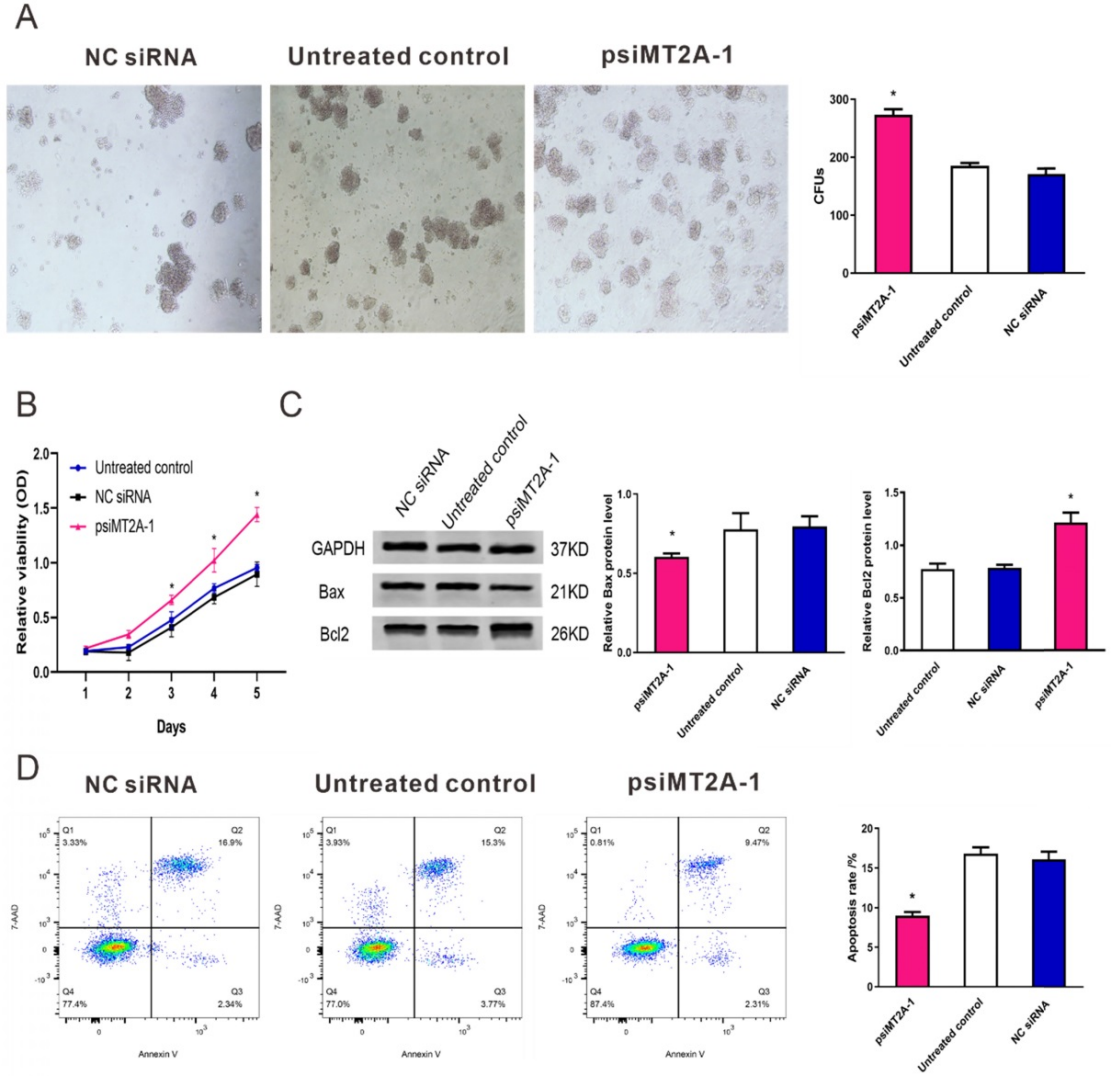
** Transfection of psiMT2A-1 induces HL60 cell proliferation.** (A) The proliferation ability of HL60 cells was measured by soft agar colony formation analysis. The number of clones in the psiMT2A-1 group was obviously increased in comparison with the untreated and the NC siRNA group. (B) CCK-8 method was used to evaluate proliferation capacity after silencing of MT2A. (C) Western blot was executed to detect Bax and BCL2 protein expression in the psiMT2A-1, NC siRNA, and untreated groups. The densitometric ratio of Bax/GAPDH and Bcl2/ GAPDH was recorded by quantity one software. Quantification evaluation of data from independent triplicate experiments(right). (D) FCM analysis of apoptosis rates of HL60 cell after MT2A knock-down. The number of independent experiment repetitions was tightly controlled at least three times(right) and represented as mean ± SD. *P < 0.05 vs. control.

To validate the cell proliferation and apoptosis after inhibition of MT2A expression, cell proliferation tests consisted of CCK-8 assay and colony formation were used to detect the HL60 cell multiplication in vitro. The result of colony formation assay indicated down-regulation of MT2A promoted the proliferation and colony formation ability of HL60 cells (Figure 2A). At the same time, there was no statistical difference between the NC siRNA and the untreated group (*P*=0.074, Figure 2A). CCK-8 assay also demonstrated that the absorbance of the psiMT2A-1 group was notably increased in a time-dependent manner on the third day after transfection of shRNA (*P* < 0.05, Figure 2B), suggesting that inhibition of MT2A expression could accelerate cellular multiplication of HL60 cells. In addition, as the proteins that regulate the progression of apoptosis, Bcl-2 and Bax were detected to validate the results of the aforementioned cell proliferation tests. Our research showed that the increased protein level of Bcl-2 and decreased expression level of Bax in the psiMT2A-1 group (*P* < 0.05, Figure 2C). Besides, to understand the unambiguous role of MT2A on the level of cell apoptosis, we also performed FCM to assess the apoptosis rate of transfected cells. And the apoptotic rate in the psiMT2A-1 group was notably lower than the NC siRNA and the untreated group (*P* < 0.05, Figure 2D), thus indicating that the decrease of MT2A expression can effectively inhibit HL60 cell apoptosis.

### 3.3. Validation of MT2A over-expression in HL60 cells

**Figure 3:**
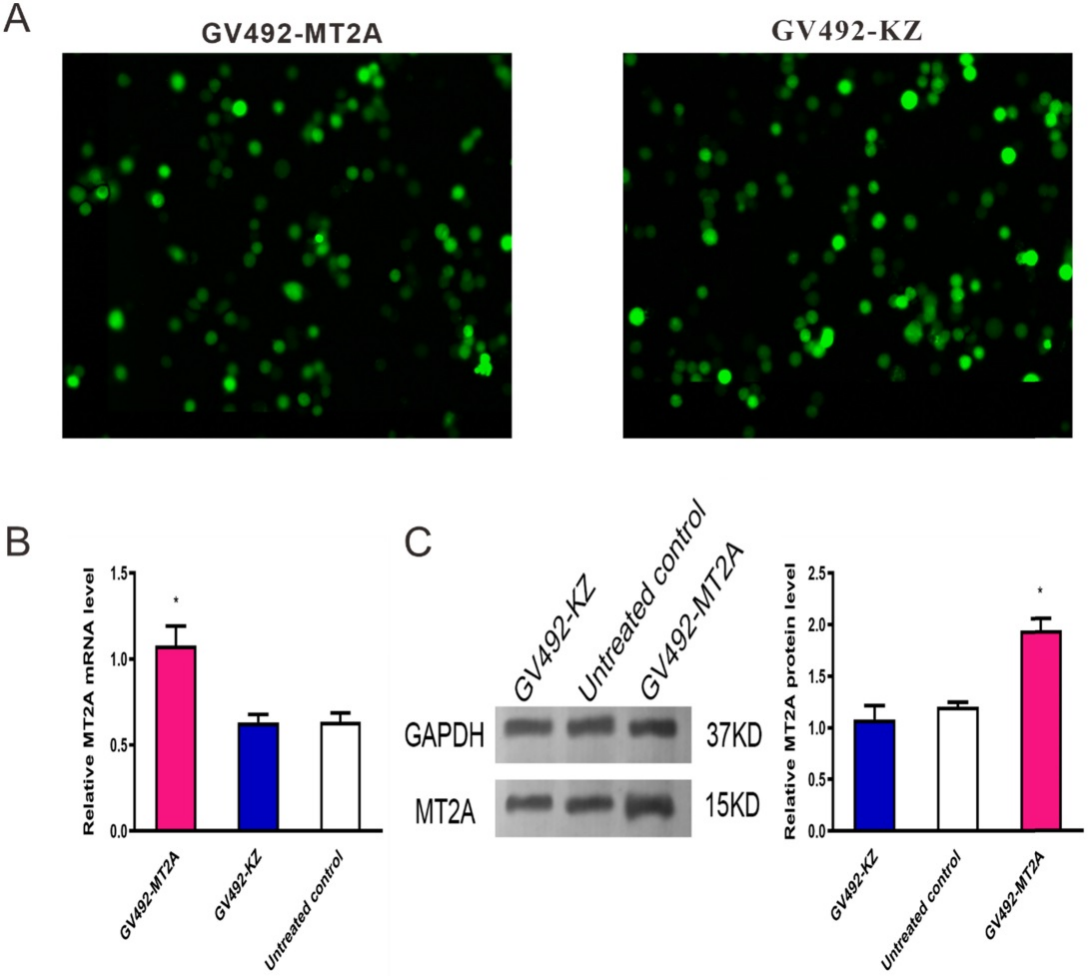
** Detection of MT2A expression in HL60 cells after infection with a lentivirus vector.** (A) Assessment of GFP expression in the GV492-MT2A and GV492-KZ group. Magnification, x200. (B) qPCR analysis of MT2A expression in each group at mRNA level after normalization with GAPDH. mRNA expression in the GV492-MT2A group was higher than in control group. (C) Western blot analysis of MT2A over-expression at the protein level. The relative MT2A protein expression level was quantified by densitometric analysis. The number of independent experiment repetitions was tightly controlled at least three times(right) and represented as mean ± SD. *P < 0.05 vs. control.

To upregulate MT2A expression, HL60 cells were infected with constructed lentivirus GV492-MT2A. Then, the percentage of GFP‑positive cells in the GV492-MT2A group (85%) and the GV492-KZ group (80%) were observed by fluorescence microscope after 72 h, indicating that the MT2A gene carried by lentivirus was successfully transferred into cells for expression (Figure 3A). In addition, qPCR (Figure 3B) and Western-blot (Figure 3C) showed that both transcription and translation levels of MT2A were markedly up-regulated, as compared with the untreated group (*P* < 0.05). In contrast, the MT2A transcription and translation levels between the GV492-KZ group and the untreated control group had no significant change (*P*=0.91, *P*=0.20).

### 3.4. Cell proliferation and apoptosis after up-regulation of MT2A

**Figure 4:**
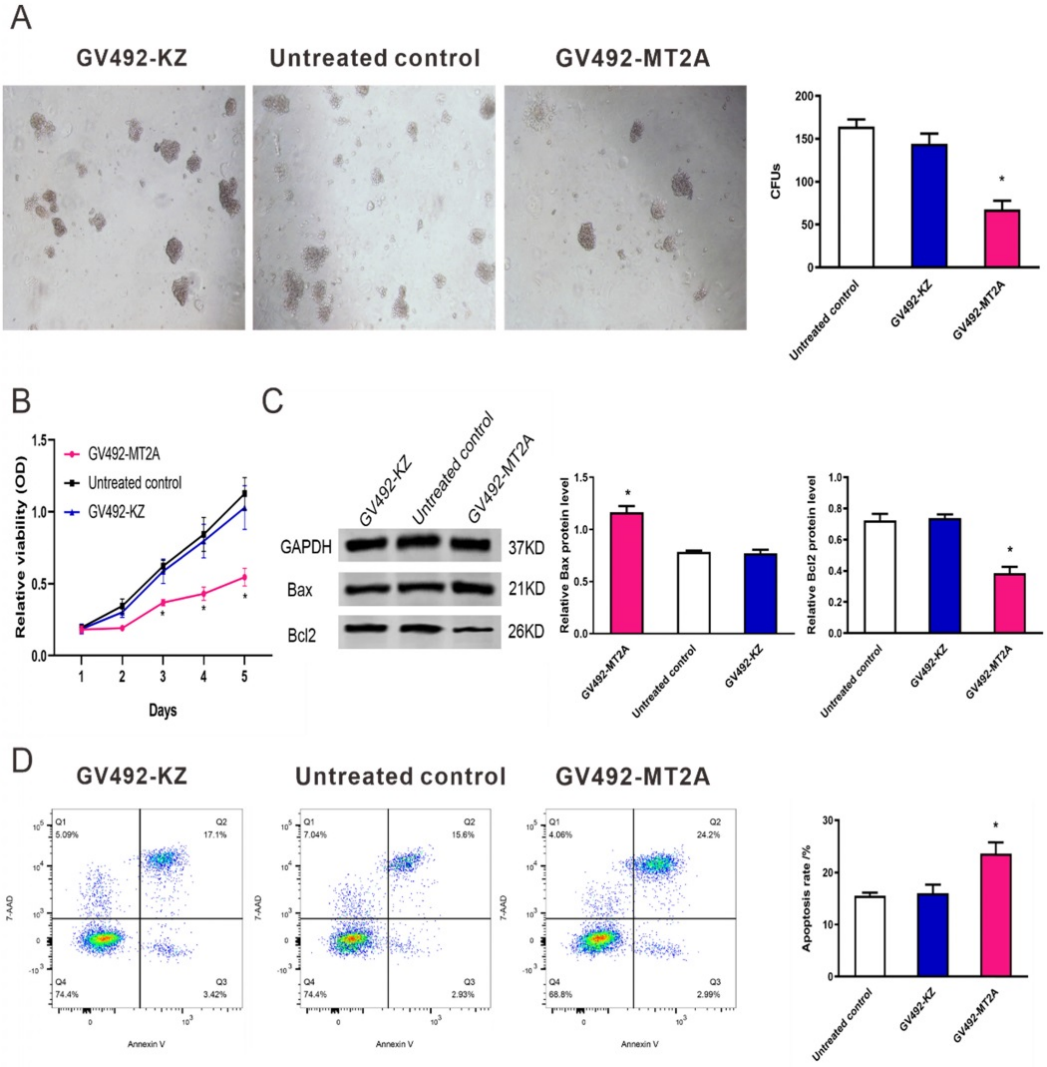
** MT2A promotes cell apoptosis of HL60 cells.** (A) The in vitro soft agar assay of HL60 cells was detected after up-regulation of MT2A. Quantification evaluation of data from independent triplicate experiments(right). (B) Cytotoxicity was examined by the CCK-8 method. The proliferation of GV492-MT2A‑infected HL60 cells was notably suppressed when compared with control. (C) Western blot analysis was used to measure the level of apoptotic proteins Bax and Bcl2. (D) The apoptosis rates of each group were evaluated using FCM analysis. The number of independent experiment repetitions was tightly controlled at least three times (right) and represented as mean ± SD. *P < 0.05 vs. control.

To evaluate the function of MT2A in HL60 cells, we up-regulated its endogenous expression by infection of GV492-MT2A. The results demonstrated that the number and size of clones in the GV492-MT2A group were reduced compared with the GV492-KZ and negative control group (*P* < 0.05, Figure 4A). The CCK-8 assay demonstrated no significant difference in HL60 cell proliferation between the GV492-KZ and the negative control group (*P >*0.05). But the proliferation level of HL60 cells in the GV492-MT2A group was prominently lower than that in the GV492-KZ and the untreated control group (*P* < 0.05, Figure 4B). Also, our findings showed that over-expression of MT2A could induce HL60 cell apoptosis by increasing Bax and inhibiting Bcl2 expression (*P* < 0.05, Figure 4C). Besides, compared with the control group, there was a significant increase in the number of apoptotic cells, suggesting that MT2A could induce the apoptosis rate of leukemia cells and reduce the cell survival rate, which is consistent with the results of CCK-8 and colony formation assay (Figure 4D).

### 3.5. Over-expression of MT2A promotes cell cycle arrest and represses NF-κB activity

**Figure 5:**
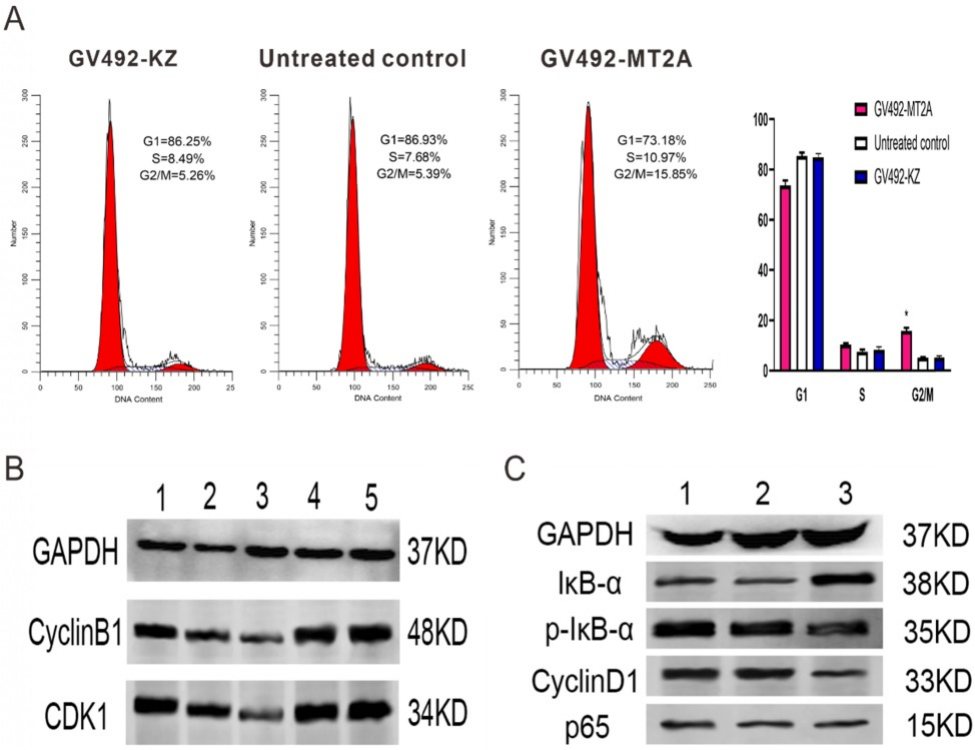
** Effects of MT2A on cell cycle distribution and NF-κB activity in HL60 human leukemia cells.** (A) FCM analysis of HL60 cell cycle changes after MT2A over-expression. Quantification evaluation of data from independent triplicate experiments(right). *P < 0.05 vs. control. (B) Western blot analysis of cyclin- regulated proteins CDK1 and CyclinB1. (1~3: 24,48,72 h after over-expression of MT2A; 4, 72 h after over-expression of MT2A in the GV492-KZ group; 5, Untreated control). (C) Up-regulation of MT2A resulted in increasing protein expression level of IκB-α and decreasing the protein expression level of p-IκB-α and CyclinD1 in HL60 cells.

FCM was carried out to detect the influence of MT2A over-expression on cell cycle changes, and Western-blot was executed to examine the CDK1 and CyclinB1 expression. FCM results displayed a notable increase in the proportion of cells at the G2/M phase of the GV492-MT2A group, suggesting that MT2A could significantly induce the cycle arrest of HL60 cells and inhibit cell proliferation (Figure 5A). The expression levels of CyclinB1 and CDK1 proteins gradually decreased compared with the control group (Figure 5B). Furthermore, the expression of IκB-α was significantly increased, while p-IκB-α and CyclinD1 were prominently decreased after MT2A over-expression and no noticeable change was shown in the expression level of P65 protein. The results showed that MT2A could repress NF-κB activity by increasing IκB-α protein expression (Figure 5C).

## 4. Discussion

AML is a malignant clonal blood disease with unclear pathogenesis. At present, it is generally believed that AML may be triggered by multiple genetic mutations, which will result in abnormal proliferation of hematopoietic cells by activating specific signaling pathways in vivo, thus gaining cell proliferation and survival advantages. It may also involve the arrest of hematopoietic differentiation induced by changes in certain transcription factors 20. As the highest proportion of adult acute leukemia types, the morbidity of AML is mounting up each year, which severely threatens susceptible populations 21. Although complete remission (CR) is generally achieved in most sufferers after chemoradiotherapy, a reasonably large portion of sufferers eventually experience recurrence or refractory recurrence, suggesting that new AML treatment strategies have not been met 22. On the other hand, the results of combination hematopoietic stem cell transplantation (HSCT) with chemotherapeutic drugs are not always satisfactory.

What's more, only ~50% of the long-term survival rate was reported in initially diagnosed patients with AML (≤60 years). Even only ~10% of the long-term survival rate existed in older AML patients 23, 24. Therefore, it is of great importance to develop new diagnosis and treatment methods for leukemia and even to explore the molecular pathogenesis of AML patients.

As the primary subtype of MTs, the MT2A gene is abnormally expressed in leukemia cells and is closely related to cell differentiation 25, 26. Multiple studies have shown that MT2A expression is vital for the development of cancer. On the one hand, MT2A is a pro-oncogene in breast cancer 27, prostate cancer 28, large B cell lymphoma 29, and adrenocortical cancer 30, and closely related with unfavorable prognosis of numerous malignant tumors. On the other hand, the decreased expression level of MT2A in gastric cancer 31, liver cancer 32, and thyroid cancer 33 are negatively correlated with cancer mortality. These results suggest that abnormal regulation of MT2A is involved in the development of tumors. However, the expression and mechanism of MT2A in AML remain unclear. Therefore, we detected the effects of MT2A gene over-expression or down-expression on HL60 cell proliferation and apoptosis and the related molecular mechanisms, to explore the possibility of MT2A as a therapeutic target biomarker for AML.

In this study, the expression level of Bax was notably inhibited, and Bcl2 was significantly up-regulated owing to down-regulation of MT2A expression, suggesting that MT2A will induce HL60 cell apoptosis by regulating the dynamic changes of Bax and Bcl2 proteins and participate in the occurrence and development of AML through the apoptotic signal network. Importantly, apoptosis is the spontaneous and orderly death of cells, which is controlled by a large family of apoptosis-related proteins in order to respond to cellular stress. The process of cellular suicide is strongly influenced by the pathophysiological mechanisms of damaged or redundant cells 34. According to the functions of apoptosis-related proteins, the Bcl-2 family is grouped into apoptosis-promoting proteins (BAX, BAD, BID, NOXA, PUMA, BMF, BIK) and apoptosis-inhibiting proteins (Bcl-2, Bcl-XL, Bcl-W) 35. The increasing levels of Bcl-2 can effectively restrain cell apoptosis, and cause abnormal cell proliferation and cell malignant transformation 36. When stimulated by cell death signals, Bax changed cellular localization from cytoplasm to the mitochondrial membrane to motivate cell apoptosis 37. However, further experiments are needed to verify the signaling pathway through which down-regulated MT2A expression promotes HL60 cell proliferation and whether the same phenomenon exists in other AML cell lines. Precisely, cell cycle regulation is accomplished by a series of essential cyclins. Mutations or changes in expression levels of these regulatory factors will lead to changes in cell cycle regulation, resulting in increased cell proliferation ability, reduced differentiation, loss of original cell functions, and eventually the development of tumor cells 38. As a crucial checkpoint except for G1/S, Cyclin B1 and CDK1 are mainly responsible for regulating the change of the G2/M phase 39. In our current study, the cells were effectively blocked in the G2/M phase after MT2A over-expression and accompanied by decreased expression of CyclinB1 and CDK1. It was suggested that MT2A could regulate the content of cycle-regulating proteins and lead to cell cycle disorders.

In fact, previous studies have shown that excessive activation of NF-κB can inhibit its original tumor suppressor function and promote the invasiveness of tumor cells 40. Specifically, NF-κB is present in almost all eukaryotic cells, which acts as a vital protein complex to respond to various stimuli from outside the body 41. As a Rel family transcription factor, NF-κB consists of five members, including c-Rel, Rel-A, Rel-B, NF-κB1, and NF-κB2 42. Besides, NF-κB is thought to function as a regulating factor that acts an essential role in cell proliferation and survival of various cancers 43. Normally, NF-κB binds to the IκB as a complex at rest state. However, in the presence of cytokines, IκB dissociates from the NF-κB/ IκB complex, further promoting p65 forms an active dimer and activates the expression of many genes, among them genes encoding Cyclin D1 and Bcl-2, a process that acts a stimulating effect on the development of malignant cells 44.

In addition, the NF-κB pathway is abnormally activated in hematopoietic diseases (multiple myeloma and AML) accompanied by a wide range of inflammatory factors, which build a terrific tumor microenvironment to promote the development of blood tumors 45, 46. Therefore, we believe that the hyperactivated NF-κB signaling pathway is related to the abnormal proliferation of AML. Besides, our results showed that MT2A over-expression can effectively inhibit the expression of enssential proteins in the NF-κB signaling pathway and reduce the invasive ability of tumor cells 31, 47. However, the specific mechanism in leukemia remains unclear.

Here, the level of IκB-α in the NF-κB pathway was significantly increased after up-regulation of MT2A. In contrast, the levels of p-IκB-α and CyclinD1 were notably decreased, suggesting that MT2A can inhibit the inappropriate pathway activation of NF-κB by mediating the increased expression of IκB-α in AML-derived HL60 cells. These research findings proved previous studies and demonstrated that the NF-κB signaling pathway could be a negative regulator of apoptosis and performed to the therapeutic target of AML. Nevertheless, whether MT2A can regulate the NF-κB signaling pathway in the process of influencing the HL60 cell process to enhance the sensitivity of chemotherapy drugs will be the focus of research in the following clinical treatment.

## Conclusion

In summary, our study demonstrates that up-regulation of MT2A can decrease the proliferation ability of HL60 cells and increase the number of apoptotic cells by affecting the changes of NF-κB pathway. Also, this study will also provide a new idea and experimental basis for further understanding MT2A as the specific molecular diagnostic targets in the therapy of AML.
